# How to improve eyewitness testimony research: theoretical and methodological concerns about experiments on the impact of emotions on memory performance

**DOI:** 10.1007/s00426-021-01488-4

**Published:** 2021-02-19

**Authors:** Kaja Głomb

**Affiliations:** Institute of Applied Psychology, Faculty of Management and Social Communication, Jagiellonian University in Kraków, ul. Łojasiewicza 4, 30-348 Kraków, Poland

## Abstract

The purpose of this paper is to present crucial shortcomings of research into eyewitness testimony. It presents the state-of-the-art of research on the relationship between emotions and memory performance. In addition, it addresses contradictions and concerns about previous studies. Despite the declarations of consensus on the role of emotions in memory coding and retrieving, there are as many studies suggesting that emotional events are better remembered than neutral ones, as there are reports that show the opposite. Therefore, by indicating the theoretical and methodological limitations of previous studies, this paper advocates a more rigorous approach to the investigation of emotions and their impact on the quality and quantity of testimony. It also provides a framework for inquiry that allows better comparisons between studies and results, and may help to build a more comprehensive theory of the effects of emotion on memory

## Introduction

Eyewitnesses often make mistakes, misreport and misidentify; thus, some of them are unreliable sources of information. There is hardly a textbook on the psychology of eyewitness testimony that suggests otherwise. After more than 40 years of research on memory relating to criminal events, this notion is increasingly accepted outside the field of academic psychology and influences legal proceedings and decisions.

However, with growing awareness of human memory limitations and its consequences for eyewitness testimony comes a discussion about the status of knowledge based on laboratory research. For many decades, almost every empirical article in the field discusses shortcomings of research. Yet, they rarely include practical guidelines on how to improve research. As a consequence of questionable methodological basis, there is no shortage of controversy over conflicting results. This seems particularly evident in the case of research on the influence of emotions on eyewitness testimony, which only superficially seems to have been resolved.

The purpose of this paper is to draw attention to the shortcomings of laboratory research concerning the effect of emotions on memory performance, and to analyse the consequences for comprehension and the application of empirical findings. Moreover, it will address them by suggesting a theoretical and methodological framework that may benefit research and serve as general guidelines for improving the quality of studies. In consequence, it will be easier to draw solid, well-grounded conclusions about the role of emotions in memory performance.

It should be noted that this paper is not a systematic review of the issue. Readers interested in more general knowledge on the application of eyewitness testimony research may refer to other papers available in the literature (e.g. Chae, 2010; Kassin, Tubb, Hosch, & Memon, 2001; Wagstaff, MaCveigh, Boston, Scott, Brunas-Wagstaff, & Cole, 2003).

## What do we know about emotions and eyewitness testimony?

Eyewitness testimony is a legal term that refers to an account of a crime given by an individual who has seen or been involved in that event. It often includes not only bystanders but also victims. Eyewitness testimony can have different forms, from a description of an event or a perpetrator, to the identification of suspects or important objects such as weapons or vehicles. For many decades, research on eyewitness testimony has focused on the identification of individual, situational, environmental, and system variables that impact the quality and quantity of testimony. Consequently, we acknowledge now that eyewitness evidence may not be as reliable as we used to think.

Among estimator variables, a category including factors that discount or augment the credibility of eyewitnesses (Wells, 1978), which can be only considered *post factum,* emotions are often discussed. Crime is a stressful experience for victims and for witnesses. Even an accidental bystander may become emotionally involved. The common suspicion and distrust we have about emotions and people showing them have led us to consider them a source of memory errors. However, as we will see, studies do not always confirm this notion.

The question of how emotions impact the way criminal events are remembered was first asked by pioneers of applied psychology. One of the first experiments on the subject, described in Münsterberg's essay (1908/2009), was conducted by von Liszt at the University of Berlin, at the beginning of the twentieth century. The experiment, which simulated a life-threatening situation, showed that memories of unexpected events accompanied by fear and a sense of danger are more distorted and misreported than emotionally neutral events.

There are many more recent studies that support this claim. It is considered that stressful stimuli negatively impact memory of perpetrator characteristics, pertinent actions, and details of crime scenes. The notion is evidenced by laboratory experiments and case studies (Clifford & Hollin; 1981, Kuehn, 1974; Loftus & Burns, 1982, Southwick, Morgan, Nicolaou, & Charney, 1997, Takahashi, Itsukushima & Okabe, 2006; for a meta-analysis see Deffenbacher, Bornstein, Penrod, & McGorty, 2004). It is possible that negative emotions have detrimental effects on the reconsolidation of episodic memory through stress hormones (Schwabe & Wolf, 2010).^1^ In consequence, as Bornstein and Robicheaux (2008, p. 525) state: “the expert consensus on eyewitness memory and arousal is that in most respects, arousal exerts a negative effect on eyewitness performance”.

However, if we take a closer look at the research results, the only obvious thing is that the influence of emotions on testimony is far from obvious. There seems to be as much evidence of the negative impact of emotions on the reliability of eyewitness testimony as there is evidence to the contrary. Empirical findings suggest that memory of stressful events tends to be accurate, as evidenced by research in eyewitness testimony (e.g. Block, Greenberg & Goodman, 2009; Christiansona & Hübinette, 1993; Christianson et al., 1991; Houston, Clifford, Phillips, & Memon, 2013; Maras, Gaigg & Bowler, 2012; Smeets, Candel & Merckelbach, 2004), as well as in the more general approach (e.g. Anderson, Wais & Gabrieli, 2006; Bookbinder & Brainerd, 2017; Bradley, Greenwald, Petry & Lang, 1992; Kensinger, Garoff-Eaton & Schacter, 2007; Laney, Campbell, Heuer & Reisberg, 2004).

A good example of the difficulties in reconciling research results is the issue of the weapon focus effect (WFE), a phenomenon describing the allocation of attention resources on highly emotive, threatening objects and, in consequence, reduced ability to describe or identify an offender and other details of the event. The overall significant difference in identification accuracy in weapon-present and weapon-absent conditions has been demonstrated (Steblay, 1992; Fawcett, Russell, Peace & Christie, 2013). However, Hulse and Memon (2006) showed that, while the presence of a weapon increases arousal, it does not always inhibit recognition accuracy. Some studies also suggest that the effect may not be as general as usually presented (e.g. Carlson & Carlson, 2012).

Therefore, the only consensus we can acknowledge is on the differences in remembering emotional versus neutral stimuli. Especially as it is supported by evidence showing distinct neural patterns of information processing depending on the stimulus valence and arousal (Kensinger & Corkin, 2004).

In the light of many contradicting results, questions about their applicability outside laboratory settings, and concerns about the validity of conditions and mental states created in a lab, the debate about the current state-of-the-art of eyewitness testimony research is hardly surprising. However, acknowledgment that the research on eyewitnesses’ emotions may not reflect reality is only the starting point. The next step would be to discuss possible reasons why we are still unsure how to estimate the impact of emotions experienced by eyewitnesses on their memory performance.

## How eyewitness testimony research goes astray

Two separate issues should be considered when systematising knowledge in this field. The first concerns the way memory is studied and discussed. In the author's opinion, contradictions are essentially the result of excessive simplifications in the presentation of research results, and of too far-reaching conclusions drawn from examination of a particular memory process. It seems that the influence of emotions on memory may not be broad and general, as it is sometimes portrayed. The impact seems to vary depending on memory function under investigation (recollection and recognition), the paradigm used to test memory (free recall, structured recall, etc.) and the content of a memory (central versus peripheral details, faces versus objects, etc.). Considering those nuances in study reports should be a common practice rather than a brief element of research discussion.

The second issue concerns emotions. While analysing research papers on the relations between affective states and memory performance, one may conclude that the vast body of theoretical and empirical studies on emotions are taken into account insufficiently. Thus, this paper will discuss three major issues concerning the way emotions are addressed and investigated in eyewitness testimony research, and which may be why we face difficulties when comparing study results. These concerns involve the way emotions are (1) defined, (2) induced and (3) measured in laboratory settings.

### Defining emotions

It is a convenient truism that emotions are difficult to define. As interest in research on emotions intensified in the late 1980s, almost every major theorist developed a distinctive concept of what emotions are and how they differ from other related affective states, such as moods or attitudes. The wealth of terms and definitions should not, however, be an excuse to ignore the body of knowledge related to the theory of emotions. Unfortunately, it is common to get the impression that, in the case of forensic psychology experiments, the word “emotion” is considered self-defining—a concept that needs no further explanation.

Typically, most research investigating human emotions falls into one of two theoretical categories—discrete or dimensional. The first approach views emotions as specific affective states that can be labelled (e.g. anxiety, fear, anger, etc.), while the other describes them according to the dimensional space of their core properties—valence, arousal, and less often, dominance. Even though it cannot be said that one or the other is more or less correct, the dimensional approach is more likely to focus on the fundamental organisation of emotions (Scherer, 2005). When this approach is employed, at least two affective dimensions (arousal and valence) should also be considered. However, studies are typically focused on one or the other (Bradley, 1994).

The lack of precision in defining the area of interest is noticeable in eyewitness testimony research. A wide range of terms is used to name affective states, such as negative emotions, arousal or stress. There is often no reflection on what these terms imply, or on the theoretical methodological consequences of their use. When we look more closely at research subjects and objectives, we can see that the terms chosen for independent variables are rarely set within the theoretical framework. Furthermore, they only seemingly relate to the approaches described above. In consequence, as the concepts are used arbitrarily and thoughtlessly, some studies answer questions other than those which they ask.

#### Negative emotion

When using the term negative emotion, emphasis seems to be on the valence—the subjective meaning an individual gives to a stimulus. However, eyewitness testimony research commonly uses the term primarily to investigate the discrete emotions—fear, anger, anxiety—not the pleasant–unpleasant dimension. It serves as a category that includes basic/modal emotions that are a priori associated with unpleasant experiences. The term also seems to describe the implicit negative tone of the criminal event, not the affective state of an eyewitness per se*.*

However, a research problem formulated in this fashion entails the risk of investigating artifacts. As particular negative emotions differ in terms of their properties, function, and behavioural and physiological reactions, it is the author’s opinion they may influence memory coding in distinct ways. The category includes very different affective states (such as anger/rage and fear/anxiety), yet equating them and studying them as one may provide misleading results. For example, during experimental manipulation one subject may experience fear/anxiety, while another feels anger, with both labelled ‘negative emotion’. In quantitative studies, the impact of the first may be neutralised by the very different effect caused by the latter. Anger serves as preparedness for attack, as motivation to approach the stimulus. Fear/anxiety, on the other hand, evokes the behavioural tendency to escape or avoid the stimulus. In consequence, while the subject experiencing anger may be strongly focused on the stimulus that caused it, the other may divert their attention away from the stimulus. Thus, the inconclusive study results.

It is also reasonable to ask which of these discrete emotions (or rather, what kind of their configuration) reflect the actual experiences of eyewitnesses and can, therefore, serve as the best analogy of those experiences. Intuition suggests that, when a witness observes a situation in which someone suffers physical harm or threat to life, they first experience fear/anxiety. Seeing a verbal, non-physical attack may cause anger rather than fear. Thus, when adopting the discrete approach, it seems crucial to analyse the impact of emotions on testimony in a more nuanced way. Considering the term “negative emotion” collectively does not necessarily explain the relationship between affective state and memory, nor does it provide insight into eyewitness experience.

On the other hand, if the concept of negative emotion is used in terms of the dimensional approach, it may have different implications for research design. While drawing attention to the subjective valence of emotional experience, the next logical step would be to examine how memory is affected depending on stimulus assessment on a scale from unpleasant to pleasant. Moreover, as the psychology of eyewitness testimony is interested in unpleasant events, it would be ideal to differentiate the level of unpleasantness of stimuli by manipulating it from the least unpleasant to the most unpleasant. This approach would facilitate accurate and appropriate investigation into valence.

However, to examine how negative emotions impact memory, it is crucial to create an event that truly evokes the required emotional reaction. Given ethical constraints, laboratory experiments may not include a stimulus that evokes negative emotions—only one that does not evoke positive ones. There is also a possibility that the reaction of a subject exposed to a stimulus is not at all an emotion but a startlement or surprise, which are, for some scholars, instinctive reactions (e.g. Ekman, Friesen & Simon, 1985).

To sum up, it is suggested that, if the term negative emotion in fact applies the discrete approach, a study should examine the variations between the influence of different types of emotions on the reliability of eyewitness testimony. In addition, research into the valence dimension should focus on how experiences evaluated as unpleasant are remembered, compared to neutral and pleasant ones.

#### Arousal

Besides negative emotions, a term frequently used in eyewitness testimony research to describe an affective state is arousal*.* Arousal, along with valence, is considered a core dimension of emotional experience. The ability to evoke arousal distinguishes between emotions and other affective states, e.g. moods and attitudes, and fuels further action (Frijda, 2004). It is a psychophysiological reaction to a stimulus, representing the activation of the autonomic nervous system (ANS). Thus, a study involving arousal may examine an objectively measurable state, not only an individual's subjective assessment. It is even more important, as some studies show, that self-reporting of arousal may differ from an objective measurement of ANS activation (e.g. Chivers, Seto, Lalumiere, Laan & Grimbos 2010; Salimpoor, Benovoy, Longo, Cooperstock & Zatorre, 2009).

In eyewitness testimony, research into arousal is often (although not exclusively) associated with the weapon focus effect. Thus, even though arousal is a neutral state, it is in eyewitness testimony research sometimes defined as negative (e.g. MacLin, MacLin & Malpass, 2001, Luna & Martin-Luengo, 2018) or as having implicit negative valence as it is evoked by unpleasant (crime-related) stimuli (e.g. Hämmerer et al, 2017).

This implicit negative valence may explain why, in contrast to general research on the arousal–memory relationship that suggests increased accuracy of recollection of arousing stimuli (e.g. Bradley et al., 1992), some experiments in forensic psychology show that arousal has a negative influence on eyewitness memory (e.g. Carlson, Dias, Weatherford & Carlson, 2017). However, it is important to underline alternative explanations that focus on the differences between central versus peripheral details (e.g. Kensinger, 2009), as well as the interview procedure (e.g. Quas & Lench, 2007).

When analysing studies on arousal and eyewitness testimony, we can also notice that arousal is often treated as a nominal variable. Subjects are induced into high-arousal or low-arousal/non-arousal states. Yet, arousal as a physiological reaction can be accurately measured. Thus, research does not need to limit its inference to a reduced problem. The issue of objective measures of emotional experience is further elaborated in the subsection on emotion measurements.

To sum up, it is postulated, that research should, in terms of arousal, focus primarily on examining how different levels and patterns of ANS activity affect the processing of visual information and its coding in long-term memory. It is also important to keep in mind that arousal is fundamentally neutral, but can be evoked by stimulus with negative, neutral and positive valence.

#### Stress

The lack of precision in the use of terms in eyewitness testimony research is even more evident when we look at the term stress*.* One may feel that research on the eyewitness testimony mimics trends in society at large, in which the term is applied to describe very different mental states—anxiety, fear, frustration, fatigue, sense of overwhelming or even anger. However, in eyewitness testimony research stress seems to describe properties of stimulus rather than eyewitness affective state. It is often a threatening, violent or demanding situation in which an individual often feels fear/anxiety or experiences arousal*.* However, this stimulus-based conceptualisation of stress is questionable, as it ignores the core concept of psychological stress—individual differences in coping with stimuli.

Applying the concept of psychological stress scientifically has its theoretical connotations and methodological consequences. Established theories and models of stress usually focus on the relationship between the external demands of the stressor and an individual’s coping mechanism. One of the most prominent theories of stress, formulated by Lazarus (Lazarus & Launier, 1978), views stress as a relational concept, a form of transaction between a person and an environment. It emphasises the individual’s appraisal of the significance of the stimulus in relation to how it threatens subjectively-defined well-being, and whether available coping resources are enough to deal with this threat (Lazarus & Folkman, 1986). Therefore, the term stress should be used neither to describe a specific external stimulation (as it is not synonymous with negative stimulus) nor to define a specific pattern of emotional, physiological or behavioural reactions.

With this in mind, it is the author’s opinion that research on stress and memory should be focused on individual differences, in particular examining why, for some eyewitnesses, stressful events are remembered better, while others tend to misrepresent events and misidentify suspects.

#### Moods

Even though emotions are the focal point of the paper, the issue of chronic moods requires a brief comment, as it is also a subject of research on eyewitness testimony. However, it is crucial to understand the difference between moods and emotions. While the latter are considered short, rapidly transient and intense affective states caused by a specific internal and external stimulus, moods are harder to define in terms of specific cause. As Frijda (1994, p. 59) believes, their object is 'the world as a whole'. They are also chronic and slowly passing, thus the disposition to experience a given mood allows us to draw conclusions about an individual’s personality (Meyer & Shack, 1989).

Despite this clear distinction, many empirical studies use these concepts interchangeably. It is quite possible that arbitrary application of these terms may be a legacy of research on the mood-state dependent effect. In his classical paper on associative network theory, Bower (1981) evokes happiness and sadness, and uses the terms “emotions” and “moods”, to name those states. Since imagination guided by hypnotic suggestion was employed to induce them, mood is the term which seems more appropriate to describe the experience. Confusion also concerns the use of terms “sadness” and “happiness”, which are more often used to label discrete emotions than moods.

Thus, it is postulated to use distinctive terms for short affective states evoked in response to specific stimulus, and for chronic, general, diffuse affective state, which can be considered a disposition to experience certain emotions (mood-trait) or a long-lasting affective state (mood-state). Moreover when the mood type is taken into account, it is advised to use adjectives describing the valence of the mood, for example “depressive mood” rather than “sadness”, or “elated mood” rather than “joy” or “happiness”. As some evidence suggests that chronic moods may also influence eyewitness testimony, thus control for a dispositional mood can be also considered.

To sum up the deliberations on defining emotions, it is important to highlight the misuse of terms and concepts of emotions, with little regard to theory and achievements of research on emotions. Thus, it is postulated that a study should define the spectrum of affective experience precisely and in accordance with the chosen theoretical approach. It must go hand in hand with awareness of the consequences of that choice, imposed by the research subject and methodology. Moreover, although both approaches to study emotions have their justification in the theory of emotions, it seems that in the case of eyewitness testimony research, the most appropriate would be to combine both. On the one hand, it would allow us to indicate what feelings accompany witnesses depending on the type of crime observed (discrete approach). On the other, dimensional approach may help to determine the characteristics of the emotional stimulus which seems to be particularly threatening the reliability of the testimony.

### Inducing emotions

Another area of doubt concerning research on eyewitness testimony is related to experimental manipulation, particularly the methods of inducing affective states. In experiments conducted in psychological labs, short films or movie clips presenting a simulated crime are the most frequently employed emotive stimuli. In fewer instances, a crime is staged, making the subjects real eyewitnesses to a false event. Researchers also use slide presentations with short narratives or photographs. Other methods of inducing emotion include exposure to aversive stimuli, such as threat of an injection or mild electric shocks (see Deffenbacher et al., 2004).

In research on chronic mood and its impact on eyewitness testimony, a different approach is often employed. Moods may be induced by techniques such as hypnotic suggestion, instruction to recall past events, reading statements related to certain moods (e.g. the Velten Mood Induction Procedure) or listening to music. In other cases, a different methodological approach with quasi-experimental research and subjects recruited on the basis of their dispositional mood or mood disorders (e.g. depression) is adopted.

While focusing on emotions, the fundamental question when evaluating research design is whether such experimental manipulations are sufficient to evoke real emotions, similar to those experienced by eyewitnesses and consequently, the extent to which experimental studies can be generalised in the forensic context. As Yuille and Tollestrup (1992) argue, a typical laboratory eyewitness is a passive observer, not experiencing a sense of danger or involvement in the event. Their physical and psychological well-being is not under threat. Thus, the behaviour of witnesses in this setting is not representative of that of witnesses to actual crimes. With this in mind, one should consider what type of stimulus manipulation increases the chances of evoking real emotions, not just a declaration of emotions.

In the author’s opinion, the strength of manipulation is derived from the level of the subject’s involvement in a study. Thus, in order to induce states as similar as possible to those experienced by real eyewitnesses, the distance (both physical and psychological) between subject and stimulus should be minimised. This kind of approach is widely used in social psychology, noted for psychologically meaningful, high impact experimental manipulations (Amodio, Zinner & Harmon-Jones, 2007). However, it may be difficult to achieve through short films, presentations or photos presented on a TV or computer screen.

#### Films

Although there is evidence that films can induce emotions, and they are widely regarded as ecologically valid stimuli, they must meet several conditions. After examination of movie databases serving as archives of stimuli useful in laboratory studies (e.g. Carvalho, Leite, Galdo-Álvarez & Gonçalves, 2012; Gross & Levenson 1995), it can be concluded that the most effective ones are expressive and emotionally unambiguous. The duration of the video clip may also be meaningful. Most databases include clips that last from 40 s to less than three minutes, but Gross and Levenson’s (1995) research indicates that films longer than a few dozen seconds have a better chance of evoking emotions, especially more complex ones.^2^

The hypothetical mechanism explaining how movies can influence emotions, and in consequence beliefs, attitudes, or behaviour, is the transportation into narrative effect. This describes psychological immersion into a narrative, and its effect is particularly pronounced when the emotional state of individual pre-reading or pre-watching content is consistent with the emotional tone of the narrative (e.g. Green, Chatman & Sestir, 2012). It seems that videos capable of inducing this effect have a greater chance of evoking emotional experiences comparable to those of eyewitnesses. However, it is difficult to meet these criteria with videos lasting 30–40 s, devoid of context and lacking vivid, identifiable characters with whom a viewer can form an empathic bond, which seems to be an important component of an emotional experience of a film (Tan, 1995).

Therefore, it is postulated that, when a crime event is presented via video, it should be an engaging narrative that focuses the subject’s attention. It would also be good practice to assess the film before the experiment (in a pilot study) or after the presentation (manipulation check), to investigate its impact on at least three-dimensional space, which represents the valence of the stimulus, the intensity of subjectively perceived excitement, and the dominance (the degree to which the film was involving and allowed subjects to detach from external stimuli).

#### Virtual reality

Another way to simulate eyewitness experience in the safe and controllable setting of a laboratory is to use modern technologies. Virtual reality (VR) with high-quality equipment provides a great opportunity for research into memory, as it allows the creation of a complex and rich environment that is fully under the control of the researcher. As goggles cut off external stimuli, VR minimises the distance between the observer and the scene, which increases immersion and allows transportation into a fictional event. Moreover, as many studies show, emotional, behavioural, and social reactions of people are the same in virtual reality as in everyday life (e.g. Gamberini et al. 2015; Kozlov & Johansen, 2010; Riva et al. 2007). With today's technological capabilities and relatively inexpensive equipment, VR may serve as a substitute not only for insufficiently involving films or video clips, but also field studies, hazardous because of possible uncontrolled variables affecting the result.

This method of experimental manipulation is nothing new. It has been successfully employed in many fields, eyewitness testimony included (e.g. Kloft et al, 2020). A particularly interesting approach is to use tools that are already utilised in law enforcement training, based on real-life simulators or large-screen video projections, not a programmed, thus artificial environment (e.g. Hulse & Memon, 2006; Stanny & Johnson, 2000). Provided they use equipment at the highest technological level, they can be highly realistic and involving, and thus, simulating a real experience.

#### Staged crime/live events

It seems that the most reliable way to simulate the experience of real eyewitnesses is to expose subjects to staged crime. Even though the crime is faked, an individual may for a moment experience real emotions, thus it meets the criteria of high ecological validity. The choice of such a method seems even more appropriate in the light of the evidence of differences in testimonies concerning live and recorded events (Ihlebæk, Løve, Erik Eilertsen & Magnussen, 2003). This kind of experimental manipulation is particularly useful in group conditions, where all participants watch the same event. Considering the high demands on the sample numbers, it may be harder to reproduce an identical event for a single participant. When deciding on this type of experimental manipulation, it is crucial to consider all ethical concerns as well.

To sum up the subsection on inducing emotion in a laboratory setting, it is crucial to underline the need to simulate experiences resembling (at least to some extent) those of witnesses to the crime. This can be achieved when experimental manipulation meets several criteria: (1) the stimulus is subjectively meaningful for the subjects; (2) psychological and physical (even imagined) distance between stimulus and observer is short; (3) regardless of the chosen way of presenting the crime event, subjects find it absorbing, (4) emotions and involvement are meticulously examined. This approach avoids the risk of drawing incorrect conclusions about the relationship between emotions and memory based on ineffective stimulus manipulation, or on one that induces states other than those intended.

### Measuring emotions

The choice of theoretical background in research on the impact of emotions on eyewitness testimony should have consequences related not only to how research questions are formulated and hypotheses tested, but also to the way in which the emotions are assessed and measured.

#### Self-reports

Measures of the effects of an emotion can be generally categorised as objective (behavioural, physiological) or subjective (self-reports). The latter, in the form of quantitative questionnaires, are the most common in eyewitness testimony research. They are used primarily due to convenience, arising from the ease and speed of collecting data and the lack of additional costs. However, as is often pointed out (e.g. Amodio, Zinner & Harmon-Jones 2007; Scherer, 2005), the use of self-reports carries the risk of getting unreliable statements about the individual’s experience. Subjects may not only be unaware of their emotions, but could also intentionally or unintentionally, mislead the researcher as they try to fulfil the internalised expectations of the research.

Another concern about self-reports is related to their structure. The choice between the dimensional versus discrete approach should be reflected in a properly chosen method of assessment. When it comes to the discrete approach, some doubts may arise when the list of labels is short, forcing an individual to choose one of them, even when they are uncertain about their own emotions. For instance, Yuille and Cutshall (1986) asked eyewitnesses of real crime to assess their level of stress on a seven-point scale, and to indicate any negative effects engendered by the incident (nightmares, sleeplessness). While the question of negative consequences may be a useful counterbalance for self-reports, limiting the questionnaire to one scale may have resulted in discovering artifacts. When subjects have only one option, they feel obliged to report anything, even if they perceive the event differently than assumed by the researcher.

Thus, it is postulated, any research in eyewitness testimony which assumes a priori that the results of stimulation are negative should also give subjects sufficient options to assess a wider spectrum of emotions, positive included. The experimental situation itself can be seen as novel and interesting, and some subjects in contact with unpleasant stimuli may react with a mixture of excitement and curiosity.

Still on the issue of scale, concerns about the psychometric properties of Likert-type scales depending on rating format are also worth noting. As there is no room here for a detailed discussion, readers seeking a more advanced overview are directed to other papers focused on the issue (e.g.: Cummins & Gullone, 2000; Finstad, 2010; Leung, 2011; Weijters, Cabooter & Schillewaert, 2010). However, it should be mentioned that there is substantial evidence showing that, as the internal structure of the scale does not lose its properties regardless of the number of points, the more points a scale has, the more skewness and kurtosis is reduced. Therefore, it is often suggested not only to abandon five-point scales, but even to use 11-points scales that increase sensitivity and follow the normal distribution.

In light of this, methodological considerations may be formulated in relation to studies that rely on scales with a small point range. Hypothetically, when a four-point scale is used (e.g. Houston, Clifford, Phillips & Memon, 2013), lacking a neutral point, skewness is expected. Thus, abnormal data distribution is highly probable. Consequently, when our objective is to examine if there are differences in the assessment of emotions between conditions, we should not use parametric estimation tests unless our sample size is big enough to be resistant to non-normality. The *T* test, for instance, is invalid with small samples from non-normal distributions, and by using it we risk falsely rejecting the null hypothesis. On the other hand, non-parametric tests are underpowered to detect an effect, so they increase the risk of accepting a false null hypothesis (Conover, 1998).

Moreover, it is postulated to include the measurement of chronical affective states in research plan. As mentioned, moods can influence the formulation of testimony and, due to mood-congruency effect, enhance the impact of experimental manipulation. Thus, controlling for that variable will help us estimate the interactions between emotions and moods, as well as eliminate the disruptive impact of opposite mood on stimulus manipulation. Questionnaires that allow diagnosis of dispositional mood, affective style or mood disorder are also recommended, as they can be help controlling for individual differences.

#### Psychophysiology

Another way to assess emotions uses objective measurements of physiological correlates of affective states. Emotions are accompanied by physiological arousal, which reflects the activation of the autonomic nervous system. The most common ways to measure it are to examine changes in electrodermal (EDA/GSR), cardiovascular and respiratory activity. Each has multiple indexes, which are evidence of ANS activation and allow the conclusion that a subject is experiencing not only general arousal, but even emotions. As Kreibig’s (2010) review shows, there is convincing evidence of emotion-specific ANS activity. Thus, when adopting more than just one method to investigate autonomic response patterns, it may be useful to discuss not only the dimension of core properties of emotional experience, but also the type of discrete emotion. However, it is crucial to note that consideration of only one index is insufficient to indicate what kind of emotion is experienced. Moreover, individual differences in ANS activation should be always taken into account.

Taking into consideration the shortcomings of self-reporting, as well as the nature of emotions and their dimensions that allow us to objectively measure some aspects of emotional experience, it is postulated that, in the case of research on how emotional events are remembered, a heterophenomenological approach should be adopted. This indicates both subjective (self-reports) and objective (psychophysiological) assessment of investigated variables, which may allow us to address discrepancies in previous research. When psychophysiological measurements are included, this may provide us with information on the nature of emotional engagement, demonstrating the effectiveness of experimental manipulation. Thus, it may also serve as an additional manipulation check. On the other hand, using self-reports gives us insight into subjective experiences, personal appraisals and assessments.

## Conclusion

The purpose of this paper is to present the theoretical and methodological shortcomings of research into the relationship between emotions and eyewitness testimony. In light of contradicting results, it is crucial to indicate those areas of research that may be responsible for misrepresentation of memory performance.

The summary of the discussion on emotions and their impact on testimony constitutes a proposition of a framework that would allow better comparisons between studies and explanations of conflicting results, while structuring knowledge that will eventually provide a complete theory of the influence of emotions on eyewitness testimony. The framework consists of the following steps:

1. Defining emotions and applying an approach to study them
  Even though there are no reasons to consider the discrete approach as theoretically unsound, the advantage of the dimensional model of emotions is primarily due to the methodological consequences it imposes. When both valence and arousal are considered, the researcher is advised to apply two different methods of measurement—self-reports with a multi-point Likert-like scale to assess valence, and psychophysiological measurement to study arousal. Although there are self-report methods to measure the intensity of emotional experience (e.g. Geneva Emotion Wheel^3^), it is crucial to note that, while intensity rating may correlate to arousal, it is not the same. Thus, other methods are required to measure arousal.
2. Inducing and manipulating emotions
  To draw inferences about the impact of emotions on eyewitness testimony, it is crucial to develop an effective experimental manipulation procedure. Emotions are not induced simply because subjects are shown a crime event on video. Watching staged, fictional criminal activity is part of everyday life in our modern world. To simulate real eyewitness experience, the researcher must create conditions that increase the subject’s personal involvement. This is possible if the stimulus has meaning, absorbs attention and seems real. Thus, the best methods to induce emotions are (1) staging crimes, (2) playing virtual reality scenarios, and (3) presenting films capable of evoking transportation into the narrative effect.
3. A proper memory test
  Even though emotions are the main interest of this paper, a model framework for research must also include guidelines on how to examine witnesses' memory. Empirical findings suggest that the influence of emotions depends on memory function. Thus, model research should consider applying more than one way of testing memory performance. Moreover, ecologically valid memory tests reflect what is asked from a real eyewitness. Therefore, it is advised to simulate police procedures for interviewing eyewitnesses, such as (1) free recollection, which may allow us to answer the general question on quantity of details remembered, forgotten or distorted, (2) structured recollection, which may allow us to distinguish between central and peripheral details and how they are remembered, and (3) adequate eyewitness identification procedure, which allows us to investigate if emotions can influence recognition, based on quasi-automatic information processing.
4. Control for individual differences

Besides the main guidelines considering defining, evoking and measuring emotions, as well as memory testing, it is also advised to control for variables that may explain individual differences in regulating emotions and moods, for example personality traits or affective styles.

To sum up, this paper encourages more strictly empirical studies that ensure theoretical and ecological validity, so the research better represents eyewitness experience and can serve as its parallel. This includes (1) exploiting the legacy of the psychology of emotion and its rich theoretical background for more consistent and multifaceted research designs, (2) ensuring strong experimental manipulation that simulates the eyewitnesses’ experiences to the extent set by research ethics, and (3) adopting a heterophenomenological approach to measuring emotional experience. Meeting these postulates may help us to formulate a coherent theory on the impact of emotions on eyewitness testimony.

## Footnotes

1 However, as Payne et al. (2007) demonstrated, stress induced prior to the acquisition stage of memory enhances emotional long-term episodic memories.

2 with exception of *disgust,* which seems particularly easy to evoke, almost instantly following the stimulus.

3 The *Geneva Emotion Wheel* (Sacharin, Schlegel & Scherer, 2012) is in fact a tool that combines the discrete and dimensional approaches. Thus, it is recommended for research into eyewitness testimony. Labelling emotions may be helpful for comparing results and may allow us to better understand the combination of emotions experienced by subjects.
